# The Evolving Landscape of Infective Endocarditis: Difficult-to-Treat Resistance Bacteria and Novel Diagnostics at the Foreground

**DOI:** 10.3390/jcm14062087

**Published:** 2025-03-19

**Authors:** Vasiliki Rapti, Efthymia Giannitsioti, Nikolaos Spernovasilis, Anna-Pelagia Magiorakos, Garyfallia Poulakou

**Affiliations:** 1 Third Department of Internal Medicine, School of Medicine, National & Kapodistrian University of Athens, Sotiria General Hospital, 115 27 Athens, Greece; gpoulakou@med.uoa.gr; 2 First Department of Propaedeutic and Internal Medicine, Medical School, National & Kapodistrian University of Athens, Laiko General Hospital, 115 27 Athens, Greece; gianiemi@hotmail.com; 3 Infectious Diseases Department, German Medical Institute, 4108 Limassol, Cyprus; nikolaos.spernovasilis@goc.com.cy; 4 Independent Researcher, 115 27 Athens, Greece; magiorakosanna@gmail.com

**Keywords:** infective endocarditis, microbiology, difficult-to-treat resistance bacteria, novel diagnostics

## Abstract

Infective endocarditis (IE) is a relatively rare but potentially life-threatening disease characterized by substantial mortality and long-term sequelae among the survivors. In recent decades, a dramatic change in the profile of patients diagnosed with IE has been observed primarily in developed countries, most likely due to an aging population and a recent increase in invasive medical procedures. Nowadays, the typical IE patient is usually older, with complex comorbidities, and a history significant for cardiac disease, including degenerative heart valve disease, prosthetic valves, or cardiovascular implantable electronic devices (CIEDs). Moreover, as patient risk factors change, predisposing them to more healthcare-associated IE, the microbiology of IE is also shifting; there are growing concerns regarding the rise in the incidence of IE caused by difficult-to-treat resistance (DTR) bacteria in at-risk patients with frequent healthcare contact. The present review aims to explore the evolving landscape of IE and summarize the current knowledge on novel diagnostics to ensure timely diagnosis and outline optimal therapy for DTR bacterial IE.

## 1. Introduction

Infective endocarditis (IE) is a multisystem, severe disease with high morbidity and mortality that can have a wide range of clinical manifestations [1]. Despite its low annual incidence in the general population of approximately 3–10 per 100,000 persons, it remains a life-threatening disease that can result in substantial sequelae for survivors. Infective endocarditis is associated with an in-hospital mortality of roughly 20%, which can increase to 25–30% at 6 months [2]. The number of IE cases and deaths has increased globally in the last 30 years, which has led to a considerable economic burden compounded by costs associated with increasing lengths of hospital stays and treatment [3,4].

Historically, the primary patient risk factors for IE included rheumatic and congenital valvular heart disease [2,5]. However, today, risk factors for IE have evolved. They are increasingly associated with aging, age-related morbidities, invasive procedures, cardiac implanted devices, intravenous drug use (IDU), and immunosuppression, leading to an increasing incidence of bacterial IE, including device-related and prosthetic valve endocarditis (PVE) [2,5,6,7]. A microbiological shift has also been reported; staphylococci have now surpassed streptococci as the predominant causative agents, while the incidence of enterococci and Gram-negative bacteria is on the rise [2,5].

Healthcare-associated IE accounts for 25–30% of all IE cases and is continuously increasing [2]. Furthermore, the emergence and increased incidence of healthcare-associated infections caused by multidrug-resistant (MDR) organisms in vulnerable patients raises concerns about whether the burden of bacterial IE caused by DTR will also increase. To our knowledge, DTR bacterial IE has been poorly studied, and guidelines are not adapted to this growing IE patient group.

Herein, we attempt to provide a comprehensive overview of the emerging microbiology of IE caused by DTR Gram-positive bacteria (GPB) and Gram-negative bacteria (GNB), current therapeutic strategies, as well as diagnostic imaging techniques and molecular assays used for the early diagnosis and management of IE.

## 2. Emerging Microbiology in IE

IE can occur in native or prosthetic valves and can be healthcare-associated or community-acquired. Risk factors for IE include older age and the presence of invasive or prosthetic cardiac devices, e.g., prosthetic heart valves, pacemakers, and IDU [8].

Globally, there is a rising incidence of healthcare-associated IE caused by Staphylococci, which includes *S. aureus* and coagulase-negative *Staphylococci* (CoNS), especially methicillin-resistant strains [1,2,5]. Enterococci have surpassed streptococci as the predominant pathogen in bacterial IE in older patients and those with transcatheter aortic valve replacements (TAVRs) [9,10,11,12]. In contrast, the incidence of streptococcal IE is declining in high-income countries [12]. In this descriptive review, we examine the changing landscape of emerging resistance in GPB- and GNB-IE, which is increasingly caused by healthcare-associated bloodstream infections (BSIs) [13,14]. These BSIs and subsequent IE are caused by MDR, extensively drug-resistant (XDR) and pan-drug-resistant (PDR) bacteria, and those classified as DTR [15]. The emergence of antimicrobial resistance and the advent of MDR, XDR, PDR, and DTR bacteria is concerning because empiric therapeutic regimens are no longer effective, appropriate treatment of infections is delayed, and, in many instances, there are few or even no effective antibiotics with which to treat infections [13,15,16].

### 2.1. Antimicrobial Therapy

Antimicrobial therapy aims to eradicate the bacteria causing endocarditis. This is achieved by obtaining source control through appropriate antimicrobial treatment, considering various factors influencing the selection of the right antibiotic(s). Such factors include local and patient antimicrobial resistance patterns, the potential for the bacterium to develop resistance during treatment, difficulties in the penetration of antibiotics into the vegetation due to bacterial biofilm production, the presence of resistant bacterial subpopulations, and whether the IE is native valve endocarditis (NVE), prosthetic valve endocarditis (PVE), or involves a cardiac implantable device. Additionally, one must consider the systemic toxicity of certain antibiotics, patient antibiotic intolerance, and whether the antibiotics used are bactericidal or bacteriostatic [8,17].

### 2.2. DTR Gram-Positive IE

Among cases of IE where an etiological bacterium is identified, up to 90% are caused by Gram-positive cocci; staphylococci, streptococci, and enterococci are the most common causes [7,18]. Antimicrobial resistance does not usually pose a significant problem when the causative pathogen belongs to the streptococci group [19]. Staphylococci and enterococci have developed mechanisms that render them resistant to key antibiotics, e.g., methicillin and vancomycin, leading to ineffective treatment and poor patient outcomes. Moreover, antibiotics used for treatment are often constrained by their toxicities, making treatment even more complex [20,21,22].

#### 2.2.1. Methicillin-Resistant *S. aureus* (MRSA)

*S. aureus* is the leading cause of IE in most countries globally [7,18,23,24,25,26]. *S. aureus* can result in severe IE by causing valvular destruction, abscess formation, septic emboli, and multi-organ disease [27]. PVE occurs in 20–30% of patients with IE, and *S. aureus* is the most common bacterium involved, usually causing IE within the first two months after valve replacement (VR) [28]. Among *S. aureus* isolates and depending on regional variations and shifts in susceptibility patterns over time, 13.5- 49.3% exhibit resistance to methicillin [24,29,30,31]. Patients with MRSA IE are more likely to have a greater number of chronic comorbidities and will develop healthcare-associated IE more frequently compared to patients with methicillin-susceptible *S. aureus* (MSSA) IE [32]. Risk factors associated with MRSA IE include chronic obstructive pulmonary disease, previous invasive procedures in the 3 months before IE symptom onset, and more than 7 days between symptom onset and hospital admission [29]. In most [31,33,34,35,36] but not all reports [29,32], MRSA IE was associated with a higher likelihood of death compared to MSSA IE. As a point of interest, some studies observed an association between higher vancomycin minimum inhibitory concentration (MIC) values (≥1.5 μg/mL or ≥2 μg/mL, depending on the study) and worse patient outcomes [37,38,39,40]. However, other studies did not confirm this [27,29,41].

Several cell wall-associated virulence factors produced by *S. aureus* can mediate its attachment to cardiac valves, contributing to their destruction. Such factors include clumping factors A and B and fibronectin-binding proteins A and B, which bind to fibronectin and fibrinogen, contributing to inflammation and subsequent endothelial cell invasion [42,43]. In addition, *S. aureus* exhibits the unique ability to form biofilms very early in the course of IE, further enhancing attachment but also making it challenging to treat, since the penetration of antibiotics into biofilm is hindered [44,45]. However, *S. aureus* is particularly adept at colonizing the heart valves and surviving shear stress, whereas other common causes of bacteremia rarely result in endocarditis. This difference has not been fully elucidated [46].

From a clinical standpoint, studies have not reported any significant differences in the symptoms and signs of patients with MSSA versus MRSA IE [29,34,47,48]. However, several studies have noted that persistent bacteremia was significantly more common in patients with MRSA IE than those with MSSA IE [32,34,47,48]. The persistence of bacteremia in the case of MRSA IE may be related to the slow eradication of MRSA by vancomycin, which is currently the antibiotic of choice for MRSA IE [48,49]. This underscores that, although vancomycin is the treatment of choice for MRSA bacteremia and IE, it is not as effective as treating MSSA IE with a beta-lactam antibiotic [50]. These worse outcomes of MRSA IE compared to MSSA IE could theoretically be attributed to the lower antibacterial efficacy of vancomycin against MRSA compared to the corresponding antibacterial performance of beta-lactams against MSSA [36].

Generally, vancomycin has certain limitations that prevent it from being as effective in bacterial eradication in IE compared to other antibiotics, such as beta-lactams. Vancomycin is characterized by slow bactericidal activity and poor penetration into cardiac valvular vegetations [51]. In addition, the trough-guided dosing administration of vancomycin carries a potential risk of nephrotoxicity [49]. To circumvent this issue, vancomycin therapy can also be monitored using the “Area Under the Curve (AUC)”. Even though there may be methodological flaws in the data used to create the recommendations [52], the AUC may be beneficial due to the lower rate of kidney injury, the fewer requirements for blood sampling, and the shorter length of stay [49,53].

Depending on the circumstances and the MRSA MIC, MRSA NVE may be treated with vancomycin alone, but as in most cases of PVE, prosthetic valve and implantable device MRSA IE requires the use of vancomycin in combination with other antibiotics, such as gentamicin and rifampicin [8].

Because MRSA is resistant to multiple antibiotics, only vancomycin, daptomycin, ceftaroline, and dalbavancin are available to treat severe infections. Apart from vancomycin, daptomycin, and ceftaroline can be combined in high doses for the treatment of NVE MRSA IE, while daptomycin may be combined with fosfomycin, cloxacillin, or gentamicin plus rifampicin (the latter for PV MRSA IE) [8,54,55]. High-dose daptomycin (10–12 mg/Kg) seems to have at least the same efficacy as vancomycin in MRSA IE [56]. In comparison, it has been associated with better outcomes in cases of MRSA bacteremia with high vancomycin MICs (>1 mg/mL) [57,58]. Alternative regimens include fosfomycin plus imipenem [59], quinupristin–dalfopristin with or without beta-lactams [60], beta-lactams plus linezolid [61], high doses of trimethoprim/sulfamethoxazole plus clindamycin [62,63], and beta-lactams plus vancomycin [64]. Finally, dalbavancin may be used as sequential therapy for MRSA IE in carefully selected patients on an outpatient basis [65,66], particularly in people with barriers to the standard of care with a failure rate of 33% [67]. Data related to the use of oritavancin for MRSA IE are still scarce [68]. Most previously mentioned treatment recommendations for MRSA IE also apply to IE caused by coagulase-negative methicillin-resistant staphylococci [8].

Staphylococcal IE is unlikely to be controlled only with antibiotics due to its rapid progression and the pathogen’s ability to cause perivalvular tissue destruction and abscess formation [69,70], which is further complicated by resistance to most beta-lactams, except ceftaroline, in the case of MRSA IE. Given the virulent nature of *S. aureus,* patients with MRSA IE should be followed by cardiothoracic surgeons from the start of their diagnosis and the decision to perform surgery should be evaluated depending on factors including the persistence of bacteremia, destruction of valve, septic emboli, abscess formation [8,71,72,73].

#### 2.2.2. Vancomycin-Resistant Enterococci (VRE)

*Enterococcus* spp. are responsible for 10–15% of IE cases worldwide and are the third most common causative pathogens implicated [18,69]. More than 50 species of enterococci have been described, with *E. faecalis* and *E. faecium* being the most clinically relevant [74]. The vast majority of enterococcal endocarditis cases are caused by *E. faecalis*, while less than 10% are caused by *E. faecium* or other species [75]. Enterococci can exhibit resistance to beta-lactams due to the expression of low-affinity penicillin-binding proteins (PBPs); the vast majority of *E. faecium* are penicillin-resistant, whereas this is not the case for *E. faecalis*. *E. faecalis* is more virulent than *E. faecium*, but the latter demonstrates higher rates of resistance to vancomycin [75,76,77].

Enterococcal resistance to glycopeptides is caused by acquiring transferrable plasmids carrying a *van* gene. This significant acquired resistance phenotype renders enterococci resistant to vancomycin and is observed in approximately 50% of *E. faecium* isolates worldwide. The prevalence of VRE is higher in North America, where approximately 65% of *E. faecium* isolates are resistant to vancomycin, compared to Europe, where about 25% of isolates are resistant. In contrast, resistance to vancomycin is recorded in less than 5% of *E. faecalis* isolates globally [78].

VRE colonization occurs mainly in the gastrointestinal tract, since enterococci are part of the normal gut flora and, to a lesser extent, in the genitourinary tract and on the skin [79]. Immunosuppression, which includes solid organ transplantation (SOT) hematological malignancies and hematopoietic stem cell transplantation (HSCT), increased length of hospital stay, proximity to another colonized patient, hospitalization in a setting with a high prevalence of VRE, residency in a long-term care facility, diabetes, renal failure, and prior exposure to antibiotics such as vancomycin, cephalosporins, aminoglycosides, carbapenems, clindamycin, and metronidazole are considered risk factors for VRE colonization [80,81,82,83,84,85,86,87,88]. VRE colonization may lead to subsequent infection, including VRE IE. A recent systematic review and meta-regression analysis shows that 8% of VRE-colonized patients develop infection within 30 days [89]. Since VRE IE is uncommon, most data regarding the outcomes of VRE infections are derived from studies that included VRE bacteraemic patients, which showed higher mortality compared to patients with bacteremia caused by vancomycin-susceptible enterococcal isolates [90,91]. Notably, VRE IE caused by *E. faecium* has been linked to tricuspid valve infection, whereas VRE IE caused by *E. faecalis* is linked to mitral valve infection, the presence of a central venous catheter, and liver transplantation [92].

*Enterococci* carry many secreted and bacterial surface virulence factors, including aggregation substance, gelatinase, cytolysin, enterococcal surface protein, and hyaluronidase, which promote adherence to cells, bacterial colonization and spreading, attachment to abiotic surfaces, and biofilm production [93,94,95]. Furthermore, enterococci display a variety of intrinsic and acquired mechanisms of antimicrobial resistance, not only against vancomycin but also ampicillin, aminoglycosides, carbapenems, and, more rarely, daptomycin and linezolid [74]. A high level of resistance in aminoglycosides further restricts therapeutic options, and combination treatment is mandatory for enterococcal bacteremia and IE, at least at the start of therapy. In addition, the enterococcal genome is quite malleable. It can effectively use insertion sequences, transposons, and plasmids to continuously acquire and transmit antibiotic-resistance genes and genes that encode several virulence factors [76,96,97].

Enterococcal endocarditis usually follows a subacute clinical course, primarily as fever, weight loss, and malaise. A murmur can be noted upon physical exam [74,98]; Osler’s nodes, Roth’s spots, and petechiae are less commonly seen [74,99]. Notably, in a large multicenter prospective cohort study on IE due to *Enterococcus* spp., patients with *E. faecalis* IE had a significantly higher risk of stroke compared with patients with *E. faecium* IE [75]. Finally, colonoscopy for colorectal disease seems necessary for patients with enterococcal endocarditis and an uncertain infection source [100,101].

Antibiotic treatment options for VRE IE are limited, even though beta-lactam antibiotics may be used to treat VRE strains if they are susceptible to beta-lactams. According to the recent European IE guidelines, daptomycin in high doses is recommended as the backbone antibiotic in the antibiotic regimen and should be combined with ampicillin, ertapenem, ceftaroline, or fosfomycin to prevent the development of resistance [8]. The resistance to daptomycin of vancomycin-resistant *Enterococcus* spp. was below 1% in the SENTRY antimicrobial surveillance program [78]. However, in some European countries, resistance rates between 5–10% have been reported [102]. In the case of resistance or severe intolerance to daptomycin, linezolid and quinupristin–dalfopristin may be used as a second-line regimen [8].

Salvage treatment options for IE caused by multidrug-resistant enterococcal strains include daptomycin plus tigecycline [103,104,105], daptomycin plus chloramphenicol [106], minocycline plus chloramphenicol [107], quinupristin/dalfopristin plus high-dose ampicillin [108], and oritavancin [109]. Finally, regarding surgical treatment, the previously mentioned principles for MRSA IE also apply to VRE IE [8].

### 2.3. DTR Gram-Negative IE

#### 2.3.1. *P. aeruginosa*

IE caused by *P. aeruginosa* is rarely seen in clinical practice and occurs in a maximum of 3% of IE cases [110]. In two prospective multicenter cohort studies of 1722 and 2751 patients with IE, the overall incidence of *P. aeruginosa* IE was 0.75% and 0.4%, respectively [111,112]. In several other studies investigating non-HACEK GNB-IE, *P. aeruginosa* was found to be among the two most prevalent pathogens causing GNB-IE [113,114,115,116,117,118,119].

Historically, IE caused by *P. aeruginosa* has been associated with IDU in up to 90% of reported cases and was classified as community-acquired [120,121,122]. However, a shift towards nosocomial or healthcare-associated *P. aeruginosa* IE has been recently observed owing to the growing population at risk, such as patients with comorbidities, immunosuppression, and a history of a previous intravascular device-related procedure, such as PV replacement, central venous catheter (CVC) insertion, arteriovenous (AV) graft operation, cardiac catheterization, pacemaker insertion, and open heart surgery [110,111,112,123,124,125,126]. The first cases of *P. aeruginosa* endocarditis following TAVI are gradually emerging in the literature [123,127,128,129]. Notably, patients with cardiac devices who develop *P. aeruginosa* bacteremia seem to be at greater risk for cardiac device-related infections [129], and, in turn, *P. aeruginosa* should be carefully considered in device-related IE [110]. Lastly, the possibility of IE should be raised in patients on hemodialysis who present with persistent *P. aeruginosa* bacteremia [130].

*P. aeruginosa* endocarditis has substantial morbidity and mortality and is characterized by frequent relapses, affecting more than one-third of patients after adequate treatment [110,125]. The rate of complications reaches approximately 85% and 65% in community-acquired and healthcare-associated IE, respectively, exceeding that of *S. aureus* endocarditis [32,110]. Based on recent reports, overall mortality reaches 28.6% in community-acquired IE and 40% in healthcare-associated [110].

Some notable characteristics of *P. aeruginosa* should be considered, as they can complicate the selection of antibiotics, leading to difficult eradication of the organism. *P. aeruginosa* displays intrinsic resistance to various antibiotics and can develop resistance while on antibiotic therapy. Furthermore, it can acquire resistance genes, rendering treatment suboptimal or ineffective [131,132]. In addition, biofilm-mediated resistance and the development of a persistent subpopulation of multidrug-tolerant cells with low metabolic activity are responsible for recalcitrance and relapse of infections [131,132,133,134]. The molecular evolution of beta-lactam resistance has been documented, as well as cases of “unstable” *P. aeruginosa* endocarditis in which the isolated strain sequentially developed multidrug- resistance to diverse anti-pseudomonal beta-lactam classes through gene mutation while on therapy [123,135,136,137,138]. The above highlights the importance of repeating susceptibility testing on serial isolates when persistent bacteremia is encountered.

Antimicrobial therapy for *P. aeruginosa* endocarditis consists of six weeks of antibiotic therapy with two antipseudomonal agents from two synergistic antibacterial classes based on the susceptibility results of the isolated strain [139]. Although no large-scale studies exist that explore the efficacy of combination versus monotherapy, the vast majority of published data support combination antibiotic therapy based on the following arguments: (i) to prevent *P. aeruginosa* from developing resistance during anti-pseudomonal chemotherapy, which occurs especially if monotherapy is chosen [134], (ii) to provide potential synergy, allowing for two different mechanisms of bacterial killing, and (iii) to support the significant mortality benefit achieved by the combination of two or more antibiotics in cases of *P. aeruginosa* bacteremia [140].

The recommended therapy for *P. aeruginosa* endocarditis is to use a high-dose anti-pseudomonal beta-lactam antibiotic, such as meropenem or ceftazidime, in combination with an aminoglycoside, preferably tobramycin or amikacin, except when contraindicated, such as when patients have impaired renal function because of potential nephrotoxicity [17,141]. Alternatively, high-dose ciprofloxacin and an anti-pseudomonal beta-lactam can be administered [142]. Interestingly, delafloxacin, a novel dual-targeting fluoroquinolone approved for the treatment of both acute bacterial skin and soft tissue infections (SSTIs) and community-acquired bacterial pneumonia in adult patients [143], is characterized by an enhanced ability for intracellular and biofilm penetration, as well as increased potency in acidic environments, thus making it a promising agent for infections involving biofilm [144]. GNB isolates resistant to other fluoroquinolones were shown to retain their susceptibility to delafloxacin [145,146]. This was demonstrated in a study of *P. aeruginosa* isolates from adults with cystic fibrosis, where 33.3% isolates with intermediate resistance and 35.7% isolates resistant to ciprofloxacin were all found to be sensitive to delafloxacin [146]. Hence, delafloxacin can be a promising antimicrobial agent for treating ciprofloxacin-resistant *P. aeruginosa* isolates. Of note, antimicrobial agents should be administered at the highest possible dose, and a loading dose should be initially given for treating all GNB-IEs. Given the time-dependent bactericidal activity of beta-lactams [147], optimized administration of the antibiotic by extended or continuous infusion coupled with therapeutic drug monitoring is advocated as an essential stewardship strategy to improve clinical outcomes by achieving better concentrations in cardiac vegetations, especially at higher MICs [148,149].

Alarmingly, the prevalence of DTR *P. aeruginosa* IE has been reported as high as 7% [150]. This poses a significant challenge for the selection and effective treatment of IE caused by DTR or PDR *P. aeruginosa* strains because of limited available treatment options. Successful therapy of refractory *P. aeruginosa* endocarditis has been reported with ceftazidime/avibactam (CAZ/AVI) and ceftolozane/tazobactam-based antimicrobial therapy [138,151]. Additionally, cefiderocol is a novel siderophore cephalosporin that demonstrates antimicrobial activity against a variety of MDR bacteria and has potent in vitro activity against a wide range of GNB, including carbapenem-resistant *A. baumannii*, *P. aeruginosa*, and *S. maltophilia* [152]. The compassionate use of cefiderocol as an adjunctive treatment for XDR and metallo-beta-lactamase (MBL)-producing *P. aeruginosa* IE has been described in case reports [153,154]. In one report, a patient with XDR *P. aeruginosa* NVE was treated with colistin plus cefiderocol, resulting in the control of the patient’s persistent bacteremia after 83 days and permitting a successful valve replacement [153]. In a different report in which the patient had an MBL-producing *P. aeruginosa* IE associated with CIED IE, which was resistant to both CAZ/AVI and ceftolozane/tazobactam, it was treated with cefiderocol and imipenem. This, along with source control, contributed to the eradication of the organism [154]. There is a paucity of data on the effectiveness of the other novel antibiotics such as imipenem/relebactam and meropenem/vaborbactam against DTR *P. aeruginosa*.

There are few evidence-based recommendations for the treatment of IE by DTR *P. aeruginosa*. Combination treatment with a cephalosporin or carbapenem with an aminoglycoside seems to be an established standard against *P. aeruginosa* [141], even though there are only a few *P. aeruginosa* isolates with resistance to these classes of antibiotics [124]. In such cases, extrapolating on guidelines for treating severe infections with other XDR and PDR Gram-negatives [155], treatment should likely be with two classes of antibiotics to which the pathogen is susceptible. A prolonged course of beta-lactam antibiotics is recommended, as in other DTR infections such as BSIs and ventilator-associated pneumonia (VAP) [147,156].

A combination of surgical treatment and the administration of antibiotics has better clinical outcomes than antibiotic treatment alone in *P. aeruginosa* endocarditis, although there are no patient outcome data on therapeutic regimens specific to XDR *P. aeruginosa* strains comparing surgical treatment plus antibiotic therapy versus antibiotic therapy alone. In a published review of left-sided IE, the mortality rate in patients receiving medical treatment alone was 62% (eight of thirteen cases) compared to 31% in patients receiving surgical treatment plus antibiotic therapy [124]. Limitations of this study were the small sample size and the fact that the more severely ill patients and patients with severe comorbidities were treated with a medical approach alone. Due to the difficulty in clearing bacteremia in DTR *P. aeruginosa* IE, a combination of antibiotic therapy and surgical intervention should be considered early on [150,154].

#### 2.3.2. Enterobacterales

##### *E. coli* 

*E. coli* can frequently result in bacteremia [157]. In an international multicenter study, the burden of IE was low, accounting for 0.5% of cases [112]. In contrast, in other studies, it was higher, found to be the causative microorganism in almost one-third of non-HACEK GNB-IE [111,116,117,119] and among the most common GNB that caused IE [114,158], suggesting that the incidence of *E. coli* IE may be higher. Despite its rarity, *E. coli* IE is associated with high in-hospital mortality that reaches 21% and exceeds the fatality rates in the case of HACEK IE [112,159].

Risk factors for *E. coli* IE include age > 70, immunosuppressed women, diabetes mellitus, and implanted intravascular or cardiac devices [112,160,161,162]. However, *E. coli* IE has also been described in younger patients aged 20–40 years [158,163,164,165,166], and in the absence of comorbidities or predisposing factors [163,164,166,167]. Additionally, excessive alcohol consumption, with or without cirrhosis, has been recorded as a potent risk factor for *E. coli* endocarditis [161,168], likely resulting from bacterial transmigration of *E. coli* from the damaged mucosa of the gastrointestinal tract.

Complicated tract infections (UTIs) with *E. coli* are presumed to be the initial event leading to bacteremia in many cases, causing IE [111,160,161]. This hypothesis is supported by the findings of Akuzawa and colleagues, who found that 36% of patients with *E. coli* NVE tested positive for *E. coli* in urine culture samples [161], and the molecular analysis performed by Andrade and colleagues, which revealed that the genetic characteristics of Enterobacterales (*E. coli* and *K. pneumoniae*) isolates from patients with IE were similar to those of UTI-causing isolates [169]. Additional sources for the *E. coli* are gastrointestinal sources, as reported by Quiring et al. in a review of 10 *E. coli* PVE cases documenting a preceding gastrointestinal infection or pathology in half of the cases [170], thus highlighting the association with non-genitourinary sources of *E. coli* PVE. Hence, with the relatively high incidence of *E. coli* UTI and bacteremia in hospitalized patients, clinicians should consider including echocardiography in diagnosing patients with persistent or relapsing *E. coli* bacteremia, especially in those with implanted cardiac devices.

##### *K. pneumoniae* 

*K. pneumoniae* is an established cause of community-acquired and healthcare-associated infections. Still, despite the trend toward an increased incidence of various invasive infections, including bacteremia, it remains one of the less commonly incriminated causative pathogens for IE or implantable cardiac device infections [111,112,116,117,171].

Since *K. pneumoniae* IE is not commonly diagnosed, robust data are scarce, and data can be found in published case reports. There are data from a systematic review by Ioannou et al. of only 45 patients with *K. pneumoniae* IE, who were predominantly men with a mean age of 54.5 years whose risk factors were the presence of PV or CVCs, recent cardiac surgery, end-stage renal dysfunction (ESRD) on hemodialysis, and IDU [172]. In this meta-analysis, as with other GNB-IE, the initial bacteremia was due to a complicated UTI [111,160,169,172,173]. Infection-related mortality was as high as 18%, and the IE affecting the aortic valve was independently associated with overall mortality [172].

##### Treatment of Enterobacterales Endocarditis

Treatment recommendations for Enterobacterales endocarditis are the same as for other non-HACEK GNB-IE: a 6-week antimicrobial regimen including beta-lactams combined with an aminoglycoside or fluoroquinolone [17]. Over time, several cases of IE caused by extended-spectrum beta-lactamase (ESBL)-producing Enterobacterales and carbapenemase-producing or PDR *K. pneumoniae* have been reported [161,172,174,175,176,177,178]. Concurrently, the absence of evidence-based treatment guidelines for *K. pneumoniae* endocarditis [8], along with the high rate of antimicrobial resistance that *K. pneumoniae* exhibits nowadays and the limited treatment options in the case of XDR or PDR strains [179,180], render the treatment of IE a challenge for the clinicians. Therefore, most of the reported *K. pneumoniae* endocarditis cases have been treated according to antimicrobial susceptibility testing reports of the cultured isolates, with or without surgery, leading to successful bacteremia clearance that reached 85% in susceptible non-hypervirulent strains [178].

Carbapenems, in combination with aminoglycosides, are recommended as first-line treatment of ESBL- and ceftriaxone-resistant Enterobacterales endocarditis [129,181]. Resistance to last-resort carbapenems in *K. pneumoniae* is mainly mediated by the production of beta-lactamases, such as Klebsiella pneumoniae carbapenemase (KPC), New Delhi metallo-beta-lactamase (NDM), Verona integron-encoded metallo-beta-lactamase (VIM) and oxacillinase (OXA)-48-like type enzymes [179]. Hence, endocarditis caused by carbapenem-resistant Enterobacterales (CRE) can be managed with one of the following antimicrobial regimens: (i) colistin and gentamicin combination [182], (ii) tigecycline and colistin combination based on their synergistic or additive effect against several MDR Enterobacterales isolates [177,179,183], and (iii) novel New Delhi metallo-beta-lactamase-lactamase inhibitors or cephalosporins [179,180]. According to the Infectious Diseases Society of America (IDSA) guidelines on CRE treatment, the use of ceftazidime/avibactam (CAZ/AVI) with aztreonam (ATM) or cefiderocol monotherapy is recommended for MBL-producers (NDM, VIM, or IMP) and either CAZ/AVI or cefiderocol monotherapy for OXA-48-like producers [13]. Second-line options include tigecycline, eravacycline, colistin, and fosfomycin but there are few indications [13].

So far, CAZ/AVI has been efficiently used in carbapenem-resistant *K. pneumoniae* endocarditis [184] and CAZ/AVI with ATM in PDR *K. pneumoniae* PVE [178]. In addition, the aztreonam/avibactam combination appears to be a promising option against MBL-producing Enterobacterales [180]. At the same time, the synergy of CAZ/AVI plus ATM and meropenem-vaborbactam plus ATM were shown to have similar in vitro activity against Enterobacterales producing NDM and non-OXA-48-like serine beta-lactamases, suggesting that meropenem-vaborbactam can be an alternative therapeutic option [185]. Similarly to other infections with DTR Enterobacterales, such as BSIs and VAP, the authors would recommend treatment for IE to be with two antibiotics effective against the bacteria and high-dose, prolonged administration of beta-lactams.

#### 2.3.3. *A. baumannii*–*A. calcoaceticus* Complex

*A. baumannii* has been designated to be of great clinical significance due to its association with a wide range of infectious diseases, including bacteremia, and a “red alert” pathogen owing to its extensive antibiotic resistance profile [186]. Predisposing risk factors for *A. baumannii* infection include prolonged (>90 days) hospital stay, advanced age, comorbidities, immunosuppression, major trauma, invasive procedures, previous antimicrobial therapy administration, presence of indwelling catheters, and mechanical ventilation [187].

*Acinetobacter* spp. IE is a rare clinical entity, primarily occurring in hospitalized patients with underlying factors [111,171,188,189,190]. Its exact incidence has yet to be elucidated, as most documented data arise from case reports. According to a systematic review of 35 studies, *A. baumannii*–*A. calcoaceticus* complex was the most frequently identified species, with *A. baumannii* accounting for one-third of *Acinetobacter* endocarditis cases [189]. The clinical course is characterized by an abrupt onset, aggressive clinical course, and high mortality despite effective antimicrobial therapy and surgical intervention [188,189]. The overall mortality is 39.1% for the *A. baumannii*–*A. calcoaceticus* complex [189]. Notably, *Acinetobacter* NVE is more likely to be fatal compared with PVE due to delayed diagnosis because of a low index of suspicion, resulting in delayed treatment [191].

Clinicians remain challenged by treating severe infections caused by *Acinetobacter* spp. Unfortunately, there is no simple answer to treating DTR and PDR strains. Generally, the first-line antimicrobial therapy for *Acinetobacter* is an active beta-lactam alone (e.g., imipenem, meropenem), preferably one with a limited spectrum, administered in continuous infusion and at the highest dose [186,192].

Carbapenem-resistant *A. baumannii* (CRAB) is becoming a significant public health concern and is designated as a critical-priority bacterium for which new research and development of antibiotics are needed [193]. CRAB harbors resistance determinants to other important classes of antibiotics, such as quinolones and aminoglycosides [194]. In most reported cases of *Acinetobacter* endocarditis, isolates were characterized as DTR [190], and high rates of *A. baumannii*–*A. calcoaceticus* complex strains resistant to quinolones and beta-lactams (with a carbapenem resistance rate of 66.7%) has been recorded [189].

Until now, tigecycline and colistin have been the only effective antibiotics against DTR *Acinetobacter* [179,195]. The in vivo activity of colistin was evaluated in an experimental rabbit model of *A. baumannii* endocarditis with a strain susceptible to colistin and intermediate to imipenem. Although colistin was proven effective in treating the bacteremia, it failed to successfully eradicate the bacteria from valvular vegetations due to the poor penetration and the low maximum-drug-concentration-to-MIC ratio in the tissue [196]. Therefore, combining colistin with an antimicrobial agent with a better penetration into vegetations is deemed necessary for bacteraemic endocarditis.

In a case report, Tseng and colleagues described the successful medical treatment of MDR *A. baumannii*-associated prosthetic aortic root abscess with colistin plus meropenem. Prolonged antimicrobial therapy (12 months) was deemed necessary [197]. Treatment of IE due to MDR *A. baumannii* is based on the relative literature evidence about BSIs or other infections in critically ill patients. A recent meta-analysis of the in vitro efficacy of antibiotic combination therapy against carbapenem-resistant GNB showed high or moderate synergism for polymyxin plus rifampicin against *A. baumannii* [198]. Although this in vitro synergism did not translate to increased survival in other severe infections by XDR *A. baumannii*, a randomized controlled trial showed higher microbiological eradication rates with the combination of colistin plus rifampicin as compared to colistin alone, which in the case of IE merits consideration [199].

Other data from BSIs suggest that the colistin plus meropenem combination may be superior to others if the meropenem MIC is ≤32 mg/L, while even the dual carbapenem combination can be used when carbapenemase production occurs. Triple combinations of ampicillin-sulbactam plus tigecycline plus colistin and ampicillin-sulbactam plus meropenem plus colistin have been shown in observational studies to be efficacious against PDR *A. baumannii* infections and could represent a treatment option also in IE [200]. Prolonged infusion of high doses of meropenem (3 gr every 8 h, or even higher) and ampicillin-sulbactam (9 gr every 8 h) is advised whenever used in the clinical setting for XDR *A. baumannii* BSIs [13,180].

There is no published experience using novel antibiotics against XDR *A. baumannii* IE, namely cefiderocol, eravacycline, or durlobactam. Still, they could be the backbone of a combination treatment in the case of susceptibility.

## 3. Novel Diagnostic Tools

Initially published in 1994 [201], the Duke Criteria for the diagnosis of IE were revised in 2000 [202]. Since their debut, changes in the profile and manifestations of IE necessitated modifications of the original diagnostic criteria. In this context, the International Society for Cardiovascular Infectious Diseases (ISCVID) proposed the 2023 Duke-ISCVID IE criteria incorporating new microbiological diagnostic methods, such as the enzyme immunoassay for *Bartonella* species, polymerase chain reaction (PCR), amplicon/metagenomics sequencing, in situ hybridization and diagnostic imaging tools, such as positron emission computed tomography with ^18^F-fluorodeoxyglucose (^18^F-FDG PET/CT), and cardiac computed tomography. Intraoperative inspection was also included as a new major clinical criterion. Moreover, the list of “typical” microorganisms initially considered causative in IE was expanded to include pathogens involved in intracardiac prosthetic device IE. Additional risk factors were also highlighted, such as the presence of transcatheter valve implants, CIED, and a history of prior IE [203]. Although echocardiography and microbiological cultures remain the cornerstones of the diagnosis, herein we highlight the diagnostic role of new imaging techniques and molecular assays that have been shown to increase the diagnostic yield of IE adjunctively.

### 3.1. Magnetic Resonance Imaging (MRI)

The role of brain MRI in diagnosing IE is discussed in the current European Society of Cardiology (ESC) IE guidelines [8]. Acute ischemic lesions followed by cerebral microbleeds were the most frequent imaging findings, even in neurologically asymptomatic patients [204,205,206]. In 85% of cerebral MRIs, the diagnostic threshold of IE was upgraded, conferring an additional 5.4% and 32.1% rate of diagnostic and therapeutic modifications, respectively [207,208]. In a recent study, cerebral MRI led to the modification of surgical plans in 22% of 330 patients with IE [209]. Despite pre-operative MRI findings, valve surgery for IE was successfully performed without postoperative cerebral events [210,211]. A systematic meta-analysis of 21 studies from 1990 to 2020, which included 2133 patients with IE, demonstrated that the pooled frequencies of therapeutic and surgical plan modifications were 12.8% (95% CI, 6.5–23.7%) and 14.2% (95% CI, 8.2–23.4%), respectively [206]. MRI revealed lesions compatible with IE in up to 80% of patients, even in the absence of neurological symptoms. Therefore, brain MRI is highly recommended by the current ESC guidelines for patients with (Class I) and without (Class IIb) neurological signs as a complication of IE [8]. Moreover, MRI is the imaging test of choice for detecting vertebral osteomyelitis in patients with IE, later or during the disease [8,212].

### 3.2. PET; Computed Tomography Angiography (CTA) and Leucocyte Scintigraphy with Single Photon Emission Computed Tomography (SPECT)

^18^F-FDG PET/CT and SPECT using radiolabeled white blood cells (WBC) are non-invasive techniques that serve as additional diagnostic tools in IE where echocardiography studies are doubtful or inconclusive [213]. There are two main indications for performing ^18^F-FDG PET/CT in patients with suspected infective endocarditis: (i) detection of intracardiac infection and (ii) detection of clinically silent disseminated infectious lesions due to IE [214,215]. The PET/CT scan has resulted in the upgrading of diagnoses from “possible” to “definite” IE or even to the rejection of a previously classified “suspected IE” [216]. Furthermore, PET imaging can detect perivalvular abscesses, thus changing surgical therapeutic plans [215,216]. The TreVendo study showed that ^18^F-FDG PET/CT modified classification and/or care in 40% of the patients was more likely than in those with a noncontributing echocardiography assay [217]. The most recent meta-analysis showed 86% sensitivity and 84% specificity for ^18^F-FDG PET/CT in PVE [215]. Moreover, pre-operative FDG/PET findings closely correlated to the intraoperative cardiac lesions compatible with IE, further supporting the diagnostic value of the assay [218]. As a whole-body evaluation, the PET/CT scan has excellent accuracy in detecting extracardiac septic emboli in patients with suspected endocarditis [216,219,220]. However, despite its high specificity (84–98%), the sensitivity of the PET/CT scan is poor for NVE (36%) and cardiac device-related lead infections (65%) but relatively high for PVE (86%) and cardiac device-related pocket infections (93%) [214,215,221,222]. Prior cardiac surgery may cause false-positive FDG uptake and should be interpreted with caution [223]. Overall, ESC guidelines recommend an early ^18^F-FDG PET/CT scan for the confirmation of suspected PVE (Class IB) [8], as it also establishes the diagnosis in cases of negative blood cultures and inconclusive echocardiography results and before severe structural damage occurs [223,224]. ^18^F-FDG PET/CT is predictive of major cardiac events and new events in PVE, or if applied for monitoring after medical treatment, it can confirm the resolution of previous lesions and IE relapses [215,225,226,227]. A negative follow-up assay was compatible with a lack of IE relapses [228].

Specific issues related to PET/CT scans include the patients’ preparation protocol scan acquisition, imaging reconstruction, subsequent analysis, and clinical interpretation, all of which can affect the test’s diagnostic accuracy [214,215,229]. A dual time-point FDG/PET or ECG-gated FDG PET significantly improved the detectability of IE and the sensitivity of the assay [225,230]. In contrast, new experimental probes targeting bacterial proteins improve specificity [231].

Multi-detector computed tomography angiography CTA (MDCTA) offers high-resolution anatomical information and demonstrates 100% sensitivity and specificity in detecting perivalvular complications in NVE [232]. In PVE, the combination of ^18^F-FDG PET with CTA yielded even better diagnostic performance than ^18^F-FDG PET alone (91% sensitivity, 90.6% specificity, 92.8% positive, and 88.3% negative prognostic values) with a substantial reduction of doubtful cases (from 20% to 8%, *p* < 0.0001) and a 20% upgrade of possible IE [213]. Therefore, CTA is highly recommended by current ESC guidelines for (i) confirming a possible NVE (Class IB) and (ii) the diagnosis of perivalvular complications in both NVE and PVE, especially in cases of inconclusive echocardiography results (Class IB) [8].

Leucocyte scintigraphy, along with SPECT/CT, is a complex radio nuclear assay requiring a high index of experience in the interpretation of results. However, as it demonstrated high sensitivity in detecting perivalvular infection in PVE and pacemaker IE, in persisting diagnostic uncertainty [232,233], it is included in the current diagnostic IE guidelines (Class IIaC) [8].

### 3.3. Molecular Identification of Causative Agent in IE

Implementing molecular techniques to rapidly identify pathogens and their susceptibility patterns in patients with bacteremia and sepsis facilitates prompt diagnosis and early selection of appropriate antimicrobial therapy [234]. These techniques can also be applied to the diagnosis of IE, as they shorten the time from the positivity of blood cultures (BC) to the identification of the pathogens and their antimicrobial susceptibility [235]. Blood-culture-negative IE (BCNIE) accounts for 10–20% of cases of IE [1], however, as conventional microbiological cultures are non-informative in antibiotic pre-treated patients and in cases of non-cultivable miscellaneous pathogens, e.g., *Coxiella burnetii*, *Bartonella* spp., *Mycoplasma* spp., *Thropheryma whipplei* [235]. Organism-specific primers and broad-range bacterial PCR followed by sequencing are now available as laboratory-developed tests (LDTs). A PCR of the valve was diagnostic in 92% vs. 36% of BC and 30% of serology tests in patients with *Bartonella* IE [236]. Molecular detection of *C. burnetii* in valves or blood improved the diagnosis in patients with BCNIE [237]. In a large, multicenter study that evaluated broad-range 16S rDNA polymerase chain reaction (PCR) assay for the detection of the causative microbe in culture-proven and culture-negative cases of IE in explanted cardiac valves of patients, the sensitivity was 67%, specificity 91%, the positive predictive value (PPV) 96%, and the negative predictive value (NPV) 46% [238]. Due to the relative abundance of bacterial DNA in valve tissue versus blood, testing cardiac valve tissue with broad-range plus sequencing organism-specific PCR assays is more sensitive than testing blood or serology [239,240]. Therefore, current guidelines suggest that broad-range PCR and targeted PCR, such as 16S ribonucleic acid (RNA), should be applied to blood and any intraoperative cardiac tissue [8]. Restrictions in the interpretation of the results are due to the differing patient populations and assay designs and the rarity of the disease [8]. Metagenomics by Next Generation Sequencing (NGS) has been successfully applied to the valves of patients with IE, but this method has yet to be refined [8,241]. A significantly higher diagnostic accuracy of NGS on excised heart valves compared to blood and valve cultures had been assessed [242], whereas mNGS achieved better performance with a sensitivity, specificity, AUC at 0.859, 0.727, and 0.793, respectively, which further increased when combined with blood-cultures [243]. Recently, the molecular imaging technique fluorescence in situ hybridization (FISH) combined with 16S rRNA-gene PCR and sequencing (FISHseq) was applied in IE valves, thus increasing the diagnostic yield by 35% [244].

Additionally, novel imaging and laboratory techniques serve as adjuvant tools for diagnosis, such as (i) modern three-dimensional echocardiography (both transthoracic and transesophageal echocardiography) and (ii) blood and tissue cultures with routine application of matrix-assisted laser desorption ionization-time-of-flight mass spectrometry, which allows early identification of pathogens and their antimicrobial susceptibility. Current guidelines strongly support these [8].

## 4. Conclusions

**Figure 1:**
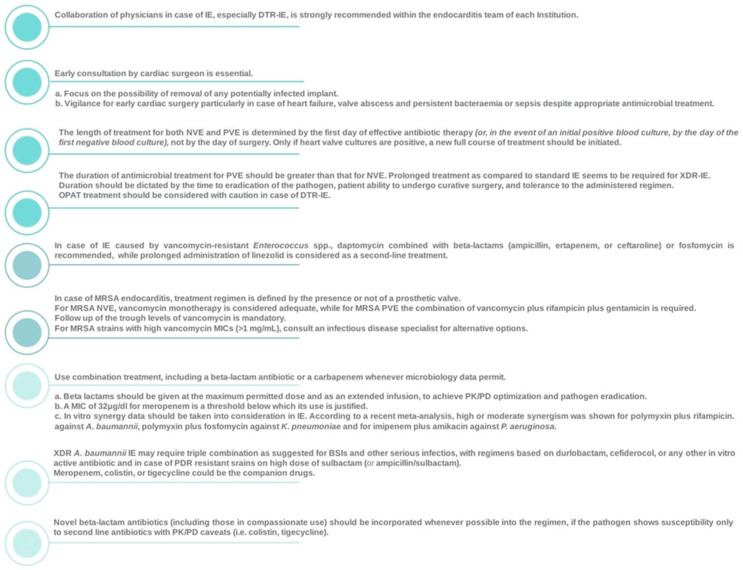
Main pillars of therapy success in case of IE. *DTR*: difficult-to-treat resistance; *IE*: infective endocarditis; *MIC*: minimum inhibitory concentration; *MRSA*: methicillin-resistant *S. aureus*; *NVE*: native valve endocarditis; *OPAT*: outpatient parenteral antibiotic therapy; *PD*: pharmacodynamics; *PK*: pharmacokinetics; *PVE*: prosthetic valve endocarditis; *XDR*: extensively drug-resistant.

Since the initial description of IE by Sir William Osler [245], scientific and technological advancements have led to changes in risk factors, microbiology, diagnosis, and treatment associated with this disease. Despite the remarkable progress in medical and surgical therapies, neither the incidence nor the mortality has decreased. The IE paradigm has changed, and new aspects of IE pathogenesis and epidemiology have emerged. The main pillars of successful management of IE in the era of difficult-to-treat resistance are illustrated in Figure 1.

Critical issues of concern are the expanding risk factors, the growing list of causative bacteria, and the emergence of MDR, XDR, PDR, and DTR resistance profiles, which lead to limited availability of therapeutic options. On the other hand, questions arise about whether stable patients diagnosed with MRSA or VRE IE could be managed with outpatient parenteral antibiotic therapy (OPAT), mainly due to the advent of novel glycopeptide antibiotics, or even switched to active oral antibiotic treatment [246].

In light of the growing resistance profile of the bacteria that cause IE, as well as the association of IE with healthcare settings, it is essential for hospitals and countries to perform surveillance and know their local bacterial resistance profiles. Infection control measures should be developed and adhered to in order to minimize healthcare-associated bacteremia and invasive infections, especially associated with indwelling devices and invasive procedures. A high index of suspicion and the appropriate methods are required for the prompt diagnosis of IE in all populations. Lastly, to correctly manage patients with IE, it is essential to establish a multidisciplinary endocarditis team for the management of patients with IE—something that has been recommended by the ESC and American College of Cardiology/American Heart Association [8,247,248] and should be the norm in most institutions. Specialists with direct involvement in diagnostic and therapeutic processes, such as cardiologists, cardiovascular surgeons, cardiac imaging experts, infectious disease specialists, and microbiologists, in addition to other specialties, e.g., neurologists, nephrologists, and nuclear medicine specialists when needed, should be at the center of the decision-making process.

The main pillars of successful management of IE in the era of difficult-to-treat resistance, according to the authors, are illustrated in Figure 1.
